# Preventing Adverse Childhood Experiences: A Framework for Culturally Responsive Home Visitation

**DOI:** 10.1177/10436596261430091

**Published:** 2026-03-17

**Authors:** Julianne Ballard, Ifeyinwa V. Asiodu, Carol Dawson-Rose, Heather Leutwyler, Catherine M. Waters

**Affiliations:** 1 University of California, San Francisco, USA

**Keywords:** maternal/child, ACEs, community health, life course, social determinants of health

## Abstract

**Introduction:**

Adverse childhood experiences (ACEs) are associated with negative health and developmental outcomes, which can persist across generations. Home visitation is a key strategy to support families impacted by ACEs. Yet, evidence-based home visitation models may not fully capture the complexities, including culture, involved in preventing intergenerational ACEs.

**Methods:**

Using a critical narrative review approach, data were extracted from the Maternal, Infant, and Early Childhood Home Visiting program and PubMed databases related to theoretical foundations and features of 13 evidence-based home visitation models.

**Results:**

Ten theories underpinned the models, predominantly focused on behavioral correction. Nurse-led models and ACEs measurement were rare. Cultural responsiveness varied widely.

**Discussion:**

The findings highlight a gap between home visitation frameworks and the factors that influence exposure to and prevention of ACEs. The proposed Culturally Responsive Home Visitation framework integrates equity, resilience, and service integration to guide research, practice, and policy aimed at preventing intergenerational ACEs.

## Introduction

Adverse childhood experiences (ACEs) are a significant public health concern shaped by the social determinants of health (SDOH) into which a person is born and raised, including economic stability, access to quality education and housing, and broader structural and community contexts (World Health Organization, 2025). Seminal research on ACEs identified a relationship between early-life exposures to family-level abuse, neglect, and household challenges—such as parental substance use, mental illness, incarceration, divorce or separation, and witnessing domestic violence—and later-life chronic illness and premature death (Anda et al., 2006; Felitti et al., 1998). Children living in poverty and those from historically marginalized communities experience ACEs disproportionately (Crouch et al., 2019; Halfon et al., 2017; Madigan et al., 2023; Merrick et al., 2018; O’Connor et al., 2020; Walsh et al., 2019). Inequities in SDOH further exacerbate these disparities by exposing children to adversities outside the family, such as discrimination, bullying, and witnessing or experiencing community violence (Beech et al., 2021; Camacho & Clark Henderson, 2022; Giovanelli & Reynolds, 2021).

An individual’s lifetime exposure to ACEs strongly correlates with their parents’ ACEs (Schickedanz et al., 2021). This pattern has been attributed to learned maladaptive parenting behaviors (Greene et al., 2020) and to the sequelae of ACEs, such as the influence of post-traumatic stress disorder on parental responses (Narayan et al., 2021). Furthermore, emerging evidence in the field of epigenetics suggests that chronic exposure to early life stressors can biologically embed changes that influence a person’s health across the life course, with the potential for these alterations to be transmitted across generations (Conching & Thayer, 2019; Švorcová, 2023; Szyf, 2015; Yehuda & Lehrner, 2018; Zhang et al., 2022).

Collectively, this body of research highlights the intricate interplay of social, cultural, environmental, psychological, and biological factors in shaping intergenerational exposure to ACEs. These insights also underscore the need for prevention strategies, such as home visitation, that are grounded in theoretical foundations focused on addressing the complex mechanisms through which ACEs perpetuate across generations. The purpose of this article is to examine the capacity of evidence-based home visitation models to capture the complexities involved in preventing intergenerational ACEs exposure, and further propose an alternative framework informed by life course theory to guide culturally responsive nurse-led home visitation.

### Capacity of Home Visitation Models to Address Intergenerational ACEs

Home visitation aims to address childhood adversity by providing parents with education and resources to create safer, healthier environments that prevent or reduce the harmful effects of ACEs for children (Garner, 2013; Kinsey et al., 2024; Narayan et al., 2021; Schickedanz et al., 2021; Zhang et al., 2023). In the United States (U.S.), nurses comprise the majority of the workforce delivering federally funded, evidence-based home visitation models, followed by educators, paraprofessionals, and social workers (Sandstrom et al., 2020). Services are offered to families experiencing health or social vulnerabilities during pregnancy, the postpartum period, or while raising a child under the age of five (Condon, 2019). Eligibility is typically based on indicators of familial vulnerability, such as poverty, parental substance use, young maternal age, and involvement with child protective services (Finello et al., 2016; Landers, 2023; Taylor et al., 2019).

The prevalence of one or more ACEs reported by adults in 23 U.S. states was found to be 62% (Merrick et al, 2028). In contrast, ACE prevalence among mothers enrolled in home visitation programs in Wisconsin was estimated at 84% (Mersky & Lee, 2019). While home visitation has been shown to improve outcomes in maternal and infant health, child development, parenting practices, and economic self-sufficiency (Condon, 2019), its overall effectiveness in preventing ACEs is mixed (Brennan et al., 2020; Brown et al., 2023; Folger et al., 2022; Howard & Brooks-Gunn, 2009; Huling et al., 2022). In addition to imprecise measures (Howard & Brooks-Gunn, 2009), unequal access to services (Kleinman et al., 2023), and lack of clarity regarding which home visitation interventions lead to successful outcomes (Nygren et al., 2018), an underlying factor likely influencing the mixed results of home visitation in preventing ACEs is the mismatch between the theoretical framework of an home visitation model, its intervention foci, and intended outcomes (Howard & Brooks-Gunn, 2009; Segal et al., 2012).

### Alignment of Home Visitation Models and Theoretical Frameworks

The alignment between a home visitation model’s theoretical framework and its objectives ensures that phenomena are contextualized in support of interventions focused on the desired outcome. In a systematic review of home visitation models in high-income countries, the effectiveness of programs in preventing or reducing child maltreatment was strongly associated with how well theoretical frameworks aligned with objectives and services provided (Segal et al., 2012). Most established home visitation models rely on theories that conceptualize ACEs prevention largely through parental behavioral deficits, environmental stressors, or both (Finello et al., 2016; Segal et al., 2012). These frameworks do not fully account for the complex interplay of social, cultural, environmental, psychological, and biological mechanisms that contribute to ACEs exposure.

In contrast, the Life Course Health Development framework explicitly addresses these intersecting mechanisms by explaining how stressful and positive exposures shape human development, emphasizing that these exposures are unequally distributed across populations (Center for Healthier Children, Families & Communities, n.d.; Halfon et al., 2014). The Life Course Health Development framework identifies “culturally-linked factors” as a subset of connected pathways that may function as risks, protective influences, or health-promoting assets (Halfon et al., 2014, p. 350). This perspective suggests that incorporating cultural responsiveness into home visitation could strengthen protective cultural influences and enhance efforts to disrupt intergenerational ACEs.

### The Role of Nurses in Providing Culturally Responsive Home Visitation

Cultural responsiveness involves the application of strength-based approaches that recognize, honor, and respect the strengths and challenges unique to a family’s cultural identity (Giordano & Edwards, 2023). Nurse-led home visitation is uniquely positioned to advance culturally responsive home visitation due to its holistic and relational approach to care, as well as its grounding in public health principles (Goldfeld et al., 2018; Monsen, 2022). Evaluations of traditional and emerging home visitation models have demonstrated nurses’ ability to assess and respond to the social, cultural, and environmental contexts that shape family health by identifying strengths, anticipating challenges, and tailoring interventions in partnership with families (Goldfeld et al., 2019; Monsen et al., 2017). However, it is unclear whether U.S. federally funded evidence-based home visitation models fully capture the complexities of preventing intergenerational ACEs exposure and support a culturally responsive approach.

## Method

Critical narrative review methodology (Grant & Booth, 2009; Sukhera, 2022) was employed to answer the following questions: (a) To what extent do the theoretical foundations and features of evidence-based home visitation models capture the complexities of preventing intergenerational ACEs exposure? and (b) How can home visitation be reimagined to build on the strengths of existing models, address gaps, account for contextual factors, and guide inquiry and culturally responsive problem-solving to prevent intergenerational ACEs disparities across populations over the life course?

**Figure 1.**
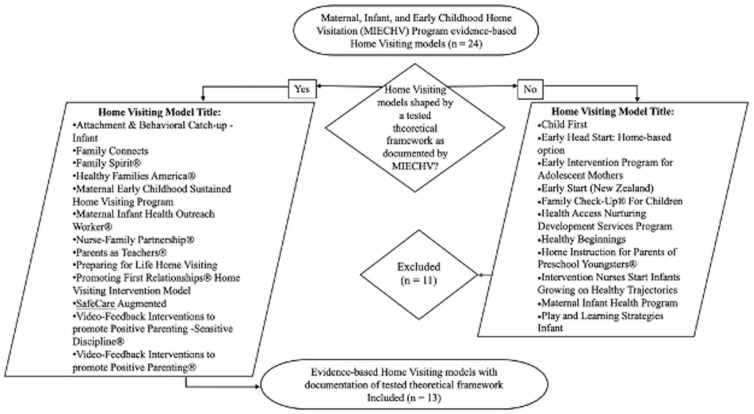
Selection of Evidence-Based Home Visitation Models. *Note.* This figure illustrates the selection process for evidence-based home visitation models approved for funding by the Maternal, Infant, and Early Childhood Home Visitation program, which were examined for inclusion in the critical narrative review.

Evidence-based home visitation models were identified through a search of the Home Visiting Evidence of Effectiveness website (n.d.) from August 2024 to February 2025; this site summarizes information specific to home visitation models eligible for U.S. federal funding and catalogs peer-reviewed literature supporting each model’s effectiveness across several domains, including reductions in child maltreatment. Of the 24 evidence-based home visitation models that met initial selection criteria, 13 were selected for review based on documentation of a tested theoretical framework (see Figure 1). Theory-specific literature was identified through a PubMed search, and literature on model features was identified through articles cataloged on the Home Visiting Evidence of Effectiveness website (n.d.) and citation chaining.

Data extracted from literature included the following model characteristics: model title, theoretical framework, presence of nurses as home visitors (yes or no), intervention focus (behavioral correction or external and internal stressors), ACEs measured (yes or no), and cultural responsiveness (none, low, moderate, or high). Data analysis followed an interpretive approach, examining the capacity of the models to address the complex factors involved in intergenerational ACEs exposure and prevention. Data synthesis involved contrasting the shortcomings of the examined models with a proposed framework informed by the Life Course Health Development framework.

## Results

Ten distinct theoretical frameworks shaped the 13 home visitation models (see Table 1). These frameworks clustered into three broad categories based on focus: (a) four theories of ecological systems, (b) four theories of development and social learning, and (c) two theories of family interpersonal and behavioral interactions and dynamics.

**Table 1.** Theoretical Foundations of Evidence-Based Home Visitation Models.

Home visitation model	Development & social learning				Family dynamics		Ecological systems			
	Attachment Theory (Bowlby, 1982)	Atribution Theory (Weiner, 1985)	Self-efficacy Theory (Bandura & Adams, 1977)	Social-learning Theory (Bandura, 1985)	Family Systems Theory (Bowen, 1978)	Coercion Theory (Patterson et al., 2010)	Ecobehavioral Model (Lutzker et al., 1998)	Ecological Theory of Human Development (Bronfenbrenner & Morris, 2007)	Ecological Framework (Jack, 2000)	Systems Theory (von Bertalanffy, 1969)
1. Attachment/Behavioral Catch-up—Infant	✓									
2. Family Connects								✓		
3. Family Spirit						✓				
4. **Healthy Families America**	✓							✓		
5. Maternal Early Childhood Sustained Home Visiting Program									✓	
6. Maternal Infant Health Outreach Worker										✓
7. **Nurse-Family Partnership**	✓		✓					✓		
8. **Parents as Teachers**		✓	✓		✓		✓	✓		
9. **Preparing for Life Home Visiting**	✓			✓				✓		
10. Promoting First RelationshipsHome Visiting Intervention Model	✓									
11. SafeCare Augmented							✓			
12. **Video-Feedback Interventions to promote Positive Parenting—Sensitive Discipline**	✓					✓				
13. **Video-Feedback Interventions to promote Positive Parenting**	✓					✓				

*Note.* Home visitation models guided by multiple frameworks are in bold text. Adapted from models eligible for the Maternal, Infant, and Early Childhood Home Visiting program (Home Visiting Evidence of Effectiveness, n.d.). In the public domain.

### Theoretical Foundations

The two most prevalent frameworks were Attachment Theory (seven models) and various ecological perspectives (eight models). Attachment Theory posits that infants seek attachment to a primary caregiver as a survival mechanism, emphasizes correcting parental behavior rather than addressing social stressors outside the family, and acknowledges that a mother’s ability to form this attachment can be disrupted by the psychological consequences of her own ACEs (Bowlby, 1982; Olds et al., 1997).

Four ecological perspectives underpinned eight of the home visitation models (see Table 1). While all four perspectives examine the relationship between individuals and their environments, they differ in focus and application. The Ecobehavioral Model emphasizes the direct modification of a person’s environment to induce specific behavioral changes (Lutzker et al., 1998). The Ecological Theory of Human Development focuses on the reciprocal relationships between a person and their environment (i.e., family, cultural mores, society, policy/legislation, etc.), and how this reciprocity shapes development across the life course (Bronfenbrenner & Morris, 2007). The Ecological Framework considers multiple layers of environmental systems, ranging from the intrapersonal to the policy level, that influence a person’s development (Jack, 2000). Systems Theory explores the interconnectedness of observable phenomena, which defy traditional scientific methods, leading to the development of biopsychosocial models that explain the nature of various factors impacting human behavior and physical health (von Bertalanffy, 1969).

Six of the 13 models (46%) were guided by two or more theories (highlighted in Table 1). The Nurse-Family Partnership (NFP) model, for example, integrated three frameworks: Attachment Theory, which informs nurses’ modeling of therapeutic relationships and efforts to reframe parental interpretations of infant behavior (Olds et al., 1997); the Ecological Theory of Human Development, which contextualizes environmental stressors (Bronfenbrenner & Morris, 2007); and Self-efficacy Theory, which supports strengths-based strategies to enhance parental confidence (Bandura & Adams, 1977). Although this combination provided a more comprehensive conceptual foundation, many interventions focused on changing parental behavior rather than addressing upstream social stressors. Two additional models, Parents as Teachers and Preparing for Life Home Visiting, were also guided by three or more theories.

### Nurse-led Home Visitation

As shown in Table 2, only three of the 13 home visitation models employed nurses as home visitors: Family Connects, Maternal Early Childhood Sustained Home Visiting program, and NFP. These nurse-led home visitation models shared an interventional focus on maternal and child health, as well as educating caregivers on positive parenting practices, but eligibility varied based on gestational stage and parity. The Maternal Early Childhood Sustained Home Visiting Program prefers, but does not require, prenatal enrollment, whereas NFP requires women to enroll early in pregnancy with their first child; both programs target vulnerable families and continue services until the child’s second birthday. In contrast, Family Connects offers postpartum support through one to three visits in a universal approach. All the nurse-led models relied on health and social assessments to guide the development of tailored interventions. Unlike nurse-led models, those led by non-nurses typically employed a more prescriptive approach, emphasizing standardized or structured curricula.

**Table 2.** Features of Evidence-Based Home Visitation Models.

Home visitation model	Nurses as home visitors	Intervention focus	ACEs measured	Cultural responsiveness
1. Attachment & Behavioral Catch-up—Infant		Behavioral correction	No	None
2. Family Connects	Yes	External and internal stressors	No	Moderate
3. Family Spirit		Behavioral correction	No	High
4. Healthy Families America		Behavioral correction	Yes	Moderate
5. Maternal Early Childhood Sustained Home Visiting Program	Yes	External and internal stressors	No	High
6. Maternal Infant Health Outreach Worker		External and internal stressors	No	High
7. Nurse-Family Partnership	Yes	Behavioral correction	No	Moderate
8. Parents as Teachers		Behavioral correction	No	High
9. Preparing for Life Home Visiting		Behavioral correction	No	Low
10. Promoting First Relationships Home Visiting Intervention Model		Behavioral correction	No	Low
SafeCare Augmented		External and internal stressors	No	Low
Video-Feedback Interventions to promote Positive Parenting -Sensitive Discipline		Behavioral correction	No	Moderate
Video-Feedback Interventions to promote Positive Parenting		Behavioral correction	No	Moderate

*Note.* ACEs = Adverse Childhood Experiences. Cultural responsiveness categorized as: None = no specificity to cultural context; Low = services provided to participants from different ethnic/racial populations; Moderate = services culturally tailored to local community and family needs; High = services with culturally-based curriculum tailored to local community and family needs. Adapted from models eligible for the Maternal, Infant, and Early Childhood Home Visiting program (Home Visiting Evidence of Effectiveness, n.d.). In the public domain.

### Intervention Focus and Adverse Childhood Experiences Measurement

Four of the 13 home visitation models employed interventions focused on addressing both external and internal family stressors, aligning with ecological theories that view childhood adversity as influenced by familial, cultural, societal, and economic factors that shape a child’s development and potential in life. The remaining nine home visitation models concentrated on correcting parental behaviors to prevent ACEs, aligning with theories that explain childhood adversity through family interpersonal dynamics and behavioral interactions, as well as development and social learning. The measurement of ACEs was not a feature in most home visitation models. Rather than using an ACEs-specific measure, childhood adversity was often assessed through proxy outcomes such as child safety, involvement with child protective services, parent-child attachment, positive parenting, parental stress, and social support (Home Visiting Evidence of Effectiveness, n.d.). Only one home visitation model, Healthy Families America, screened children for ACEs as part of a risk assessment upon entry; however, ACEs were not measured at exit or follow-up.

### Cultural Responsiveness

The cultural responsiveness of the home visitation models varied. Most home visitation models allowed for some flexibility in tailoring service delivery or curriculum to meet local community or individual family needs. However, three models demonstrated limited flexibility, referencing cultural tailoring only for specific groups or contexts. One model, Attachment and Behavioral Catch-up—Infant, made no reference to culturally responsive principles (Home Visiting Evidence of Effectiveness, n.d.).

## Discussion

The review of 13 selected evidence-based home visitation models funded by the Maternal, Infant, and Early Childhood Home Visiting program highlights both strengths and limitations in their capacity to deliver culturally responsive interventions aimed at preventing intergenerational ACE disparities. Most of the models were guided by the Attachment Theory and variations of the ecological perspective. While these frameworks provided a foundation for program design and aligned with certain intervention strategies, they did not consistently align with program objectives or intended outcomes. Interventions primarily emphasized correcting parental behaviors, often without adequately addressing SDOH stressors outside the family that can profoundly influence child development and well-being. Although home visitation has demonstrated improvements in family outcomes (Condon, 2019), the models reviewed referenced risks and protective factors only in broad terms, without specifying which interventions delivered by home visitors fostered resilience or produced measurable improvements in outcomes (Monsen et al., 2011; Nygren et al., 2018). This lack of specificity may obscure potential misalignment between theoretical frameworks and program activities, ultimately limiting effectiveness in raising awareness, preventing, and mitigating intergenerational ACEs (Howard & Brooks-Gunn, 2009; Segal et al., 2012).

Only three of the examined home visitation models were nurse-led. While literature indicates modest differences in outcomes between nurse-led and paraprofessional-led home visitation, these differences do not specifically address child maltreatment or ACEs (Olds et al., 2014; Peacock et al., 2013). Paraprofessional-led models are generally more scalable due to lower cost (Peacock et al., 2013). Nonetheless, even brief nurse-led home visitation (1 to 3 visits) has been shown to reduce child protective services investigations and emergency medical care utilization (Goodman et al., 2021), suggesting that nurses’ holistic, relational approach may provide unique value in preventing and mitigating ACEs risk.

Measurements of ACEs within these home visitation models were extremely limited. Only one model, Healthy Families America, screened children for ACEs at program entry, and no models measured ACEs as an outcome. This aligns with evidence indicating that ACEs screening tools have limited predictive sensitivity at the individual level (Anda et al., 2020; Baldwin et al., 2021; Lacey & Minnis, 2020; Meehan et al., 2022). Current guidance recommends that routine ACEs measurement in children be conducted primarily in research contexts and accompanied by broader conversations with parents about their child’s risk (Bethell et al., 2017). Reflective conversations with parents about their own ACE history may be especially valuable, as parents often request opportunities to revisit their personal ACEs screening results (Hardcastle & Bellis, 2019; Selvaraj et al., 2022). Home visitors typically have sufficient time for these conversations, with visits averaging approximately 1 hr and a mean of 28 visits annually (Hughes-Belding et al., 2019; Nygren et al., 2018), providing a feasible platform for ACEs awareness and prevention.

Given that many families served by home visitation programs are at risk for intergenerational ACEs, interventions must consider both upstream structural factors—such as poverty, inequality, and racism—and downstream individual factors, including parental stress and child maltreatment. While several evidence-based home visitation models have incorporated culturally responsive principles (Lewy, 2021), the findings of this review suggest that these principles are not consistently operationalized within program objectives or curricula. Contextual stressors, including poverty, racial and ethnic discrimination, and fear of deportation, can activate biological and structural pathways that perpetuate intergenerational ACEs, placing additional strain on family relationships (Jones et al., 2019; Kelly-Irving & Delpierre, 2019). Addressing these complexities requires home visitation models grounded in theoretical frameworks that fully capture the social, cultural, environmental, psychological, and biological mechanisms underlying ACEs exposure. Building on these insights, we propose translating the Life Course Health Development framework into home visitation practice. This approach leverages the strengths of existing models, addresses identified gaps, and explicitly incorporates SDOH and contextual factors, which provide a roadmap for culturally responsive interventions that can more effectively prevent intergenerational ACE disparities.

### Proposing a Culturally Responsive Home Visitation Framework

**Figure 2.**
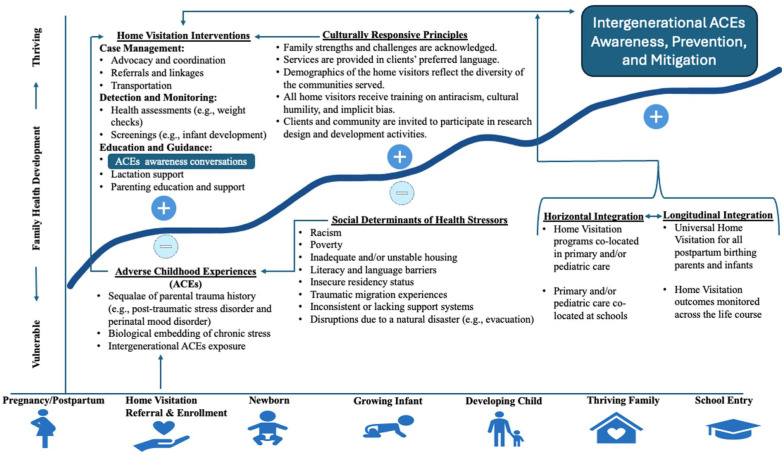
Culturally Responsive Home Visitation Framework. *Note*. This figure illustrates the life course in home visitation programs, from fetus through school entry. The wavy line denotes the family health development trajectory. Encircled plus signs denote protective factors. Encircled minus signs denote risks. Culturally responsive home visitation interventions, in coordination with other health care services, are posited to interrupt intergenerational ACEs and moderate SDOH stressors that contribute to ACEs exposure.

The Culturally Responsive Home Visitation (CRHV) framework is proposed as an alternative theoretical foundation for home visitation models. It is designed to guide culturally responsive problem-solving and research aimed at preventing intergenerational ACEs disparities across the life course. The framework incorporates core elements of nurse-led home visitation while explicitly addressing equity and the complex interplay of downstream and upstream risk and protective factors. By emphasizing resilience and developmental plasticity, the CRHV framework seeks to interrupt intergenerational ACEs exposure while supporting ongoing evaluation and refinement of culturally responsive practices across diverse populations. The five core concepts, three guiding assumptions, and three propositions of the CRHV framework are summarized in Table 3 and illustrated in Figure 2.

**Table 3.** Concepts, Assumptions, and Propositions of the Culturally Responsive Home Visitation Framework.

Component	Description
Concepts	1. **Cultural responsiveness:** Protective, equity-focused approaches that engage families by recognizing the strengths and challenges inherent in their cultural identities (Giordano & Edwards, 2023). 2. **Home visitation interventions**: Include case management, detection and monitoring, education and guidance, and ACEs awareness conversations, which differ from ACEs screening by focusing on education, reflection, and empowerment. 3. **Adverse childhood experiences**: Harmful or distressing events in childhood with long-term consequences for development, health, and well-being, often extending across generations (Anda et al., 2006; Felitti et al., 1998; Schickedanz et al., 2021). 4. **Social determinants of health**: Encompass individual and structural conditions that shape development, health, and well-being (World Health Organization, 2025). 5. **Integration of Services**: Horizontal and longitudinal coordination of services, including co-location of home visitation with primary and pediatric care and school-based programs.
Assumptions	1. **Timing matters:** Family health development thrives when interventions target sensitive periods of the life course (e.g., pregnancy and infancy). 2. **Culturally responsive interventions disrupt ACEs disparities**: Negative and positive ACEs exposures are unevenly distributed across populations. Culturally responsive home visitation can make a difference in interrupting intergenerational ACEs disparities. 3. **Equity is central**: Culturally responsive home visitation ensures that all families, regardless of background or circumstances, have opportunities to thrive.
Propositions	1. **Effectiveness of culturally responsive vs. behavior-focused interventions:** Compare culturally responsive interventions with traditional behavior-focused interventions. Outcomes may include measures of family resilience, parental engagement, and program retention, assessing both intervention timing and effectiveness. 2. **Impact of horizontal and longitudinal service integration**: Support policies promoting horizontal integration (e.g., co-locating home visitation with primary and pediatric care) and longitudinal integration (e.g., universal postpartum home visitation) to improve access, continuity, and utilization. 3. **Family and community-centered design**: Involve families and communities in designing, implementing, and evaluating services, using community-based participatory research and community advisory boards to tailor home visitation to local needs and ensure interventions reflect the lived experiences of the population.

*Note.* ACEs = Adverse childhood experiences.

#### Core Concepts

##### Cultural Responsiveness

Cultural responsiveness refers to the application of protective, equity-focused approaches that recognize the strengths and challenges inherent in a family’s cultural identities (Giordano & Edwards, 2023; Lewy, 2021). Cultural safety exemplifies one such approach successfully used by nurses to examine historical context, personal attitudes, and power dynamics, thereby fostering mutual respect and avoiding the labeling of families as inherently “at risk” (Giles et al., 2015, p. 548; Richardson et al., 2017).

##### Home Visitation Interventions

Home visitation interventions encompass case management, detection and monitoring, as well as education and guidance. These interventions are presumed to have reciprocal relationships with outcomes related to ACEs awareness, prevention, and mitigation. A novel intervention within the CRHV framework is the *ACEs awareness conversation*, which differs from ACEs screenings by focusing on education about the science of adversity, its long-term impacts, and opportunities for building family resilience.

##### Adverse Childhood Experiences

ACEs are harmful or distressing events in childhood with long-term consequences for development, health, and well-being, often extending across generations (Anda et al., 2006; Felitti et al., 1998; Schickedanz et al., 2021). These experiences may arise within the family (e.g., abuse, neglect, household challenges) or from community and broader environmental contexts (Alhowaymel et al., 2021; Beech et al., 2021; Camacho & Clark Henderson, 2022; Giovanelli & Reynolds, 2021; Kalmakis & Chandler, 2014).

##### Social Determinants of Health

SDOH encompass individual and structural conditions that shape development, health, and well-being, either positively or negatively (World Health Organization, 2025). Negative SDOH stressors increase the likelihood of ACEs exposure and help explain disproportionate burden among marginalized, low-income, and racially or ethnically minoritized children (Beech et al., 2021; Camacho & Clark Henderson, 2022).

##### Integration of Services

Integration refers to the horizontal and longitudinal coordination of services, including the co-location of home visitation with primary and pediatric care, as well as school-based programs. Such integration enhances cross-sector communication with the potential to expand participation and scalability of home visitation (Shaw et al., 2021)

#### Guiding Assumptions

##### Timing Matters

Family health development is optimized when interventions target sensitive life course periods, such as pregnancy, infancy, and adolescence, during which positive or negative exposures exert heightened influence as posited in the Life Course Health Development framework (Halfon et al., 2014).

##### Culturally Responsive Interventions Disrupt ACEs Disparities

Negative and positive ACEs exposures are unevenly distributed across populations. Negative exposures shaped by SDOH stressors impede development, whereas protective factors delivered through culturally responsive interventions mitigate these risks.

##### Equity Is Central

Equity is central to culturally responsive home visitation. Ensuring that all families, regardless of background or circumstances, have opportunities to thrive requires addressing disparities in SDOH early in the life course, which can yield long-term improvements in family health and well-being over many generations (Jones et al., 2019; Larson et al., 2018).

#### Propositions

To evaluate and test the CRHV framework, three propositions are offered to guide research, practice, and policy. These propositions highlight key areas for investigation and system-level redesign to improve utilization of home visitation services and address the limited available evidence on the impact of policy on ACEs prevention (Matjasko et al., 2022).

##### Effectiveness of Culturally Responsive vs. Behavior-Focused Interventions

The CRHV framework can be applied to compare culturally responsive interventions, such as ACEs awareness conversations, language-concordant services, and home visitors’ application of cultural safety, with traditional behavior-focused interventions. Outcomes may include measures of family resilience, parental engagement, and program retention across developmental stages, supporting assessment of both intervention timing and effectiveness.

##### Impact of Horizontal and Longitudinal Service Integration

The CRHV framework supports policies promoting horizontal integration (e.g., co-locating home visitation with primary and pediatric care) and longitudinal integration (e.g., universal postpartum home visitation). These strategies encourage the development of shared data systems to track cross-sector ACEs prevention efforts throughout the life course, including culturally responsive home visitation.

##### Family and Community-Centered Design

The CRHV framework emphasizes the involvement of families and communities in designing, implementing, and evaluating services. Approaches such as community-based participatory research and community advisory boards can tailor home visitation to local needs, ensuring that interventions reflect the lived experiences of the population. This approach reimagines home visitation as a system co-created with the populations it serves, providing a foundation for testing both its efficacy in a community trial and its effectiveness in real-world settings.

In summary, the core concepts, assumptions, and propositions of the CRHV framework outline a comprehensive and equity-centered approach to re-envisioning home visitation as a catalyst for interrupting intergenerational ACEs. The framework offers a pathway for strengthening family health development across the life course by integrating culturally responsive practices during sensitive periods of development, addressing upstream SDOH stressors, and elevating the voices of families and communities. As a practice, research, and policy guide, the CRHV framework invites ongoing refinement and evaluation to advance culturally grounded home visitation models that promote equity in ACEs prevention globally.

#### Limitations

This narrative review examined 13 of the 24 evidence-based home visitation models available for U.S. federal funding; therefore, the findings may not fully represent the breadth of traditional, community-developed, or emerging home visitation models used nationally or globally. In addition, implementing the culturally responsive practices outlined in the proposed CRHV framework may be resource-intensive, requiring access to qualified translators, a diverse and well-trained workforce, and strong partnerships with community organizations to ensure the availability, accessibility, and sustainable funding of follow-up services. Finally, the proposed framework warrants continued conceptual refinement and empirical testing. Advancing the CRHV framework will require sustained scholarship, including exploratory work and rigorous testing of its propositions across varied populations and practice settings.

## Conclusion

The proposed CRHV framework, viewed through the lens of the life course perspective, is recommended as a guide for home visitation practitioners, researchers, educators, leaders, and policymakers in addressing inequities and disparities in childhood adversity and the SDOH across diverse populations and communities over generations. The propositions within this equity-focused framework require longitudinal, intergenerational testing to examine the complex interplay between downstream and upstream risks and protective factors. Identifying which specific home visitation interventions foster resilience and plasticity to prevent intergenerational ACEs exposure is key to better aligning evidence-based home visitation with its theoretical foundations, thereby enhancing the likelihood of achieving desired outcomes. We recommend ongoing dialogue and evaluation to help refine culturally responsive home visitation strategies that can effectively disrupt childhood adversity on a global scale.

## References

[bibr1-10436596261430091] Alhowaymel F. Kalmakis K. Jacelon C. (2021). Developing the concept of adverse childhood experiences: A global perspective. Journal of Pediatric Nursing, 56, 18–23. 10.1016/j.pedn.2020.10.004 33181368

[bibr2-10436596261430091] Anda R. F. Felitti V. J. Bremner J. D. Walker J. D. Whitfield C. Perry B. D. Dube S. R. Giles W. H. (2006). The enduring effects of abuse and related adverse experiences in childhood. A convergence of evidence from neurobiology and epidemiology. European Archives of Psychiatry and Clinical Neuroscience, 256(3), 174–186. 10.1007/s00406-005-0624-4 16311898 PMC3232061

[bibr3-10436596261430091] Anda R. F. Porter L. E. Brown D. W. (2020). Inside the adverse childhood experience score: Strengths, limitations, and misapplications. American Journal of Preventive Medicine, 59(2), 293–295. 10.1016/j.amepre.2020.01.009 32222260

[bibr4-10436596261430091] Baldwin J. R. Caspi A. Meehan A. J. Ambler A. Arseneault L. Fisher H. L. Harrington H. Matthews T. Odgers C. L. Poulton R. Ramrakha S. Moffitt T. E. Danese A. (2021). Population vs individual prediction of poor health from results of adverse childhood experiences screening. Archives of Pediatrics & Adolescent Medicine, 175(4), 385–393. 10.1001/jamapediatrics.2020.5602 PMC7835926 33492366

[bibr5-10436596261430091] Bandura A. (1985). Model of causality in social learning theory. In Mahoney M. J. Freeman A. (Eds.), Cognition and psychotherapy (pp. 81–99). Springer US. 10.1007/978-1-4684-7562-3_3

[bibr6-10436596261430091] Bandura A. Adams N. E. (1977). Analysis of self-efficacy theory of behavioral change. Cognitive Therapy and Research, 1(4), 287–310. 10.1007/BF01663995

[bibr7-10436596261430091] Beech B. M. Ford C. Thorpe R. J. Bruce M. A. Norris K. C. (2021). Poverty, racism, and the public health crisis in America. Frontiers in Public Health, 9, Article 699049. 10.3389/fpubh.2021.699049 PMC8450438 34552904

[bibr8-10436596261430091] Bethell C. D. Carle A. Hudziak J. Gombojav N. Powers K. Wade R. Braveman P. (2017). Methods to assess adverse childhood experiences of children and families: Toward approaches to promote child well-being in policy and practice. Academic Pediatrics, 17(7Supplement), S51–S69. 10.1016/j.acap.2017.04.161 PMC6035880 28865661

[bibr9-10436596261430091] Bowen M. (1978). Family therapy in clinical practice. Jason Aronson.

[bibr10-10436596261430091] Bowlby J. (1982). Attachment and loss: Retrospect and prospect. The American Journal of Orthopsychiatry, 52(4), 664–678. 10.1111/j.1939-0025.1982.tb01456.x 7148988

[bibr11-10436596261430091] Brennan B. Stavas N. Scribano P. (2020). Effective prevention of ACEs. In Asmundson G. J. G. Afifi T. O. (Eds.), Adverse Childhood Experiences (pp. 233–264). Academic Press. 10.1016/B978-0-12-816065-7.00012-4

[bibr12-10436596261430091] Bronfenbrenner U. Morris P. A. (2007). The bioecological model of human development. In Damon W. Lerner R.M. (Eds.), Handbook of Child Psychology (6^th^ ed., pp. 793–828). John Wiley & Sons. 10.1002/9780470147658.chpsy0114

[bibr13-10436596261430091] Brown S. M. McConnell L. Zelaya A. Doran M. Swarr V. (2023). Tailored nurse support program promoting positive parenting and family preservation. Nursing Research, 72(4), E164. 10.1097/NNR.0000000000000662 PMC10415074 37104683

[bibr14-10436596261430091] Camacho S. Clark Henderson S. (2022). The social determinants of adverse childhood experiences: An intersectional analysis of place, access to resources, and compounding effects. International Journal of Environmental Research and Public Health, 19(17), 10670. 10.3390/ijerph191710670 PMC9518506 36078386

[bibr15-10436596261430091] Center for Healthier Children, Families & Communities. (n.d). Life course health development. https://healthychild.ucla.edu/science/key-concepts/life-course-health-development

[bibr16-10436596261430091] Conching A. K. S. Thayer Z. (2019). Biological pathways for historical trauma to affect health: A conceptual model focusing on epigenetic modifications. Social Science & Medicine, 1982, 23074–23082. 10.1016/j.socscimed.2019.04.001 30986608

[bibr17-10436596261430091] Condon E. M. (2019). Maternal, infant, and early childhood home visiting: A call for a paradigm shift in states’ approaches to funding. Policy, Politics & Nursing Practice, 20(1), 28–40. 10.1177/1527154419829439 PMC6600820 30791813

[bibr18-10436596261430091] Crouch E. Probst J. C. Radcliff E. Bennett K. J. McKinney S. H. (2019). Prevalence of adverse childhood experiences (ACEs) among US children. Child Abuse & Neglect, 92, 209–218. 10.1016/j.chiabu.2019.04.010 31003066

[bibr19-10436596261430091] Felitti V. J. Anda R. F. Nordenberg D. Williamson D. F. Spitz A. M. Edwards V. Koss M. P. Marks J. S. (1998). Relationship of childhood abuse and household dysfunction to many of the leading causes of death in adults: The Adverse Childhood Experiences (ACE) Study. American Journal of Preventive Medicine, 14(4), 245–258. 10.1016/S0749-3797(98)00017-8 9635069

[bibr20-10436596261430091] Finello K. M. Terteryan A. Riewerts R. J. (2016). Home visiting programs: What the primary care clinician should know. Current Problems in Pediatric and Adolescent Health Care, 46(4), 101–125. 10.1016/j.cppeds.2015.12.011 26872870

[bibr21-10436596261430091] Folger A. T. Nidey N. Ding L. Ji H. Yolton K. Ammerman R. T. Bowers K. A. (2022). Association between maternal adverse childhood experiences and neonatal SCG5 DNA methylation: Effect modification by prenatal home visiting. American Journal of Epidemiology, 191(4), 636–645. 10.1093/aje/kwab270 34791022 PMC9077120

[bibr22-10436596261430091] Garner A. S. (2013). Home visiting and the biology of toxic stress: Opportunities to address early childhood adversity. Pediatrics, 132(Suppl. 2), S65–S73. 10.1542/peds.2013-1021D 24187125

[bibr23-10436596261430091] Giles A. R. Hognestad S. Brooks L. A. (2015). The need for cultural safety in injury prevention. Public Health Nursing, 32(5), 543–549. 10.1111/phn.12210 26105082

[bibr24-10436596261430091] Giordano S. Edwards J. (2023). Enhancing cultural responsiveness in social service agencies (OPRE Report # 2023-338, Prepared by Insight Policy Research). U.S. Department of Health and Human Services, Administration for Children and Families, Office of Planning, Research, and Evaluation.

[bibr25-10436596261430091] Giovanelli A. Reynolds A. J. (2021). Adverse childhood experiences in a low-income black cohort: The importance of context. Preventive Medicine, 148, Article 106557. 10.1016/j.ypmed.2021.106557 PMC8594423 33857559

[bibr26-10436596261430091] Goldfeld S. Price A. Kemp L. (2018). Designing, testing, and implementing a sustainable nurse home visiting program: right@home. Annals of the New York Academy of Sciences, 1419(1), 141–159. 10.1111/nyas.13688 29791738

[bibr27-10436596261430091] Goldfeld S. Price A. Smith C. Bruce T. Bryson H. Mensah F. Orsini F. Gold L. Hiscock H. Bishop L. Smith A. Perlen S. Kemp L. (2019). Nurse home visiting for families experiencing adversity: A randomized trial. Pediatrics, 143(1), Article e20181206. 10.1542/peds.2018-1206 30591616

[bibr28-10436596261430091] Goodman W. B. Dodge K. A. Bai Y. Murphy R. A. O’Donnell K. (2021). Effect of a universal postpartum nurse home visiting program on child maltreatment and emergency medical care at 5 years of age: A randomized clinical trial. Journal of the American Medical Association Network Open, 4(7), Article e2116024. 10.1001/jamanetworkopen.2021.16024 PMC8264647 34232300

[bibr29-10436596261430091] Grant M. J. Booth A. (2009). A typology of reviews: An analysis of 14 review types and associated methodologies. Health Information and Libraries Journal, 26(2), 91–108. 10.1111/j.1471-1842.2009.00848.x 19490148

[bibr30-10436596261430091] Greene C. A. Haisley L. Wallace C. Ford J. D. (2020). Intergenerational effects of childhood maltreatment: A systematic review of the parenting practices of adult survivors of childhood abuse, neglect, and violence. Clinical Psychology Review, 80, Article 101891. 10.1016/j.cpr.2020.101891 PMC7476782 32745835

[bibr31-10436596261430091] Halfon N. Larson K. Lu M. Tullis E. Russ S. (2014). Lifecourse health development: Past, present and future. Maternal and Child Health Journal, 18(2), 344–365. 10.1007/s10995-013-1346-2 23975451 PMC3890560

[bibr32-10436596261430091] Halfon N. Larson K. Son J. Lu M. Bethell C. (2017).Income inequality and the differential effect of adverse childhood experiences in U.S. children. Academic Pediatrics, 17(7), S70–S78. 10.1016/j.acap.2016.11.007 28865663

[bibr33-10436596261430091] Hardcastle K. Bellis M. (2019, May). Asking about adverse childhood experiences (Aces) in health visiting: Findings from a pilot study. Public Health Wales NHS Trust. https://phw.nhs.wales/publications/publications1/health-visitor-enquiry-about-caregivers-adverse-childhood-experiences-aces-key-learning-from-a-pilot-evaluation/

[bibr34-10436596261430091] Home Visiting Evidence of Effectiveness. (n.d). Model search. U.S. Department of Health and Human Services. https://homvee.acf.gov/models?field_miechv_eligible=1&meets-hhs=1

[bibr35-10436596261430091] Howard K. S. Brooks-Gunn J. (2009). The role of home-visiting programs in preventing child abuse and neglect. Future of Children, 19(2), 119–146. 10.1353/foc.0.0032 19719025

[bibr36-10436596261430091] Hughes-Belding K. Peterson C. A. Clucas Walter M. Rowe N. Fan L. Dooley L. J. Steffensmeier C. Wang W. Bao J. Goodman K. (2019). Quality home visits: Activities to promote meaningful interactions. Infant Mental Health Journal, 40(3), 331–342. 10.1002/imhj.21779 30951194

[bibr37-10436596261430091] Huling J. D. Austin R. R. Lu S.-C. Doran M. M. Swarr V. J. Monsen K. A. (2022). Public health nurse tailored home visiting and parenting behavior for families at risk for referral to child welfare services, Colorado: 2018–2019. American Journal of Public Health, 112(S3), S306–S313. 10.2105/AJPH.2022.306792 PMC9184901 35679563

[bibr38-10436596261430091] Jack G. (2000). Ecological influences on parenting and child development. The British Journal of Social Work, 30(6), 703–720. 10.1093/bjsw/30.6.703

[bibr39-10436596261430091] Jones N. L. Gilman S. E. Cheng T. L. Drury S. S. Hill C. V. Geronimus A. T. (2019). Life course approaches to the causes of health disparities. American Journal of Public Health, 109(Suppl. 1), S48–S55. 10.2105/AJPH.2018.304738 PMC6356123 30699022

[bibr40-10436596261430091] Kalmakis K. A. Chandler G. E. (2014). Adverse childhood experiences: Towards a clear conceptual meaning. Journal of Advanced Nursing, 70(7), 1489–1501. 10.1111/jan.12329 24329930

[bibr41-10436596261430091] Kelly-Irving M. Delpierre C. (2019). A critique of the adverse childhood experiences framework in epidemiology and public health: Uses and misuses. Social Policy and Society, 18(3), 445–456. 10.1017/S1474746419000101

[bibr42-10436596261430091] Kinsey J. La Charite J. Russ S. Schickedanz A. (2024). Perinatal interventions to prevent Adverse Childhood Experiences (ACEs): A scoping review. PLOS ONE, 19(10), Article e0307441. 10.1371/journal.pone.0307441 PMC11501017 39446908

[bibr43-10436596261430091] Kleinman R. Ayoub C. Del Grosso P. Harding J. F. Hsu R. Gaither M. Mondi-Rago C. Kalb M. O’Brien J. Roberts J. Rosen E. (2023). Understanding family engagement in home visiting: Literature synthesis (OPRE Report# 2023-004). Office of Planning, Research, and Evaluation, Administration for Children and Families, US Department of Health and Human Services. https://www.acf.hhs.gov/sites/default/files/documents/opre/hv_reach_literature_synthesis_dec2022.pdf

[bibr44-10436596261430091] Lacey R. E. Minnis H. (2020). Practitioner teview: Twenty years of research with adverse childhood experience scores—Advantages, disadvantages and applications to practice. Journal of Child Psychology and Psychiatry, 61(2), 116–130. 10.1111/jcpp.13135 31609471

[bibr45-10436596261430091] Landers P. A. (2023, August). Maternal, infant, and early childhood home visiting program. Congressional Research Service. https://crsreports.congress.gov/product/pdf/IF/IF10595

[bibr46-10436596261430091] Larson K. Russ S. A. Kahn R. S. Flores G. Goodman E. Cheng T. L. Halfon N. (2018). Health disparities: A life course health development perspective and future research directions. In Halfon N. Forrest C. B. Lerner R. M. Faustman E. M. (Eds.), Handbook of life course health development (pp. 499–520). Springer. http://www.ncbi.nlm.nih.gov/books/NBK543702/31314286

[bibr47-10436596261430091] Lewy D. (2021, October). Addressing racial and ethnic disparities in maternal and child health through home visiting programs. Center for Health Care Strategies. https://www.chcs.org/media/Addressing-Racial-Ethnic-Disparities-Maternal-Child-Health-Home-Visiting-Programs.pdf

[bibr48-10436596261430091] Lutzker J. R. Bigelow K. M. Doctor R. M. Kessler M. L. (1998). Safety, health care, and bonding within an ecobehavioral approach to treating and preventing child abuse and neglect. Journal of Family Violence, 13(2), 163–185. 10.1023/A:1022893607387

[bibr49-10436596261430091] Madigan S. Deneault A.-A. Racine N. Park J. Thiemann R. Zhu J. Dimitropoulos G. Williamson T. Fearon P. Cénat J. M. McDonald S. Devereux C. Neville R. D. (2023). Adverse childhood experiences: A meta-analysis of prevalence and moderators among half a million adults in 206 studies. World Psychiatry: Official Journal of the World Psychiatric Association (WPA), 22(3), 463–471. 10.1002/wps.21122 37713544 PMC10503911

[bibr50-10436596261430091] Matjasko J. L. Herbst J. H. Estefan L. F. (2022). Preventing adverse childhood experiences: The role of etiological, evaluation, and implementation research. American Journal of Preventive Medicine, 62(6Supplement 1), S6–S15. 10.1016/j.amepre.2021.10.024 PMC9215220 35597583

[bibr51-10436596261430091] Meehan A. Baldwin J. Lewis S. MacLeod J. Danese A. (2022). Poor individual risk classification from adverse childhood experiences screening. American Journal of Preventive Medicine, 62(3), 427–432. 10.1016/j.amepre.2021.08.008 34635382

[bibr52-10436596261430091] Merrick M. T. Ford D. C. Ports K. A. Guinn A. S. (2018). Prevalence of adverse childhood experiences from the 2011–2014 Behavioral Risk Factor Surveillance System in 23 states. Journal of the American Medical Association Pediatrics, 172(11), 1038–1044. 10.1001/jamapediatrics.2018.2537 30242348 PMC6248156

[bibr53-10436596261430091] Mersky J. P. Lee C. P. (2019). Adverse childhood experiences and poor birth outcomes in a diverse, low-income sample. BMC Pregnancy and Childbirth, 19(1), Article 387. 10.1186/s12884-019-2560-8 PMC6819344 31660899

[bibr54-10436596261430091] Monsen K. A. (2022). Trust, translation, and transparency in public health nurse family home visiting. American Journal of Public Health, 112(S3), S220–S221. 10.2105/AJPH.2022.306850 PMC9184908 35679571

[bibr55-10436596261430091] Monsen K. A. Brandt J. K. Brueshoff B. L. Chi C.-L. Mathiason M. A. Swenson S. M. Thorson D. R. (2017). Social determinants and health disparities associated with outcomes of women of childbearing age who receive public health nurse home visiting services. Journal of Obstetric, Gynecologic & Neonatal Nursing, 46(2), 292–303. 10.1016/j.jogn.2016.10.004 27998686

[bibr56-10436596261430091] Monsen K. A. Radosevich D. M. Kerr M. J. Fulkerson J. A. (2011). Public health nurses tailor interventions for families at risk. Public Health Nursing, 28(2), 119–128. 10.1111/j.1525-1446.2010.00911.x 21732966

[bibr57-10436596261430091] Narayan A. J. Lieberman A. F. Masten A. S. (2021). Intergenerational transmission and prevention of adverse childhood experiences (ACEs). Clinical Psychology Review, 85, Article 101997. 10.1016/j.cpr.2021.101997 33689982

[bibr58-10436596261430091] Nygren P. Green B. Winters K. Rockhill A. (2018). What’s happening during home visits? Exploring the relationship of home visiting content and dosage to parenting outcomes. Maternal and Child Health Journal, 22(1), 52–61. 10.1007/s10995-018-2547-5 29948763 PMC6153727

[bibr59-10436596261430091] O’Connor M. Slopen N. Becares L. Burgner D. Williams D. R. Priest N. (2020). Inequalities in the distribution of childhood adversity from birth to 11 years. Academic Pediatrics, 20(5), 609–618. 10.1016/j.acap.2019.12.004 31841661

[bibr60-10436596261430091] Olds D. Kitzman H. Cole R. Robinson J. (1997). Theoretical foundations of a program of home visitation for pregnant women and parents of young children. Journal of Community Psychology, 25(1), 9–25. 10.1002/(SICI)1520-6629(199701)25:1<9::AID-JCOP2>3.0.CO;2-V

[bibr61-10436596261430091] Olds D. L. Holmberg J. R. Donelan-McCall N. Luckey D. W. Knudtson M. D. Robinson J. (2014). Effects of home visits by paraprofessionals and by nurses on children: Follow-up of a randomized trial at ages 6 and 9 Years. Journal of the American Medical Association Pediatrics, 168(2), 114–121. 10.1001/jamapediatrics.2013.3817 24296904 PMC4217160

[bibr62-10436596261430091] Patterson G. R. Forgatch M. S. DeGarmo D. S. (2010). Cascading effects following intervention. Development and Psychopathology, 22(4), 949–970. 10.1017/S0954579410000568 20883592 PMC2965055

[bibr63-10436596261430091] Peacock S. Konrad S. Watson E. Nickel D. Muhajarine N. (2013). Effectiveness of home visiting programs on child outcomes: A systematic review. BMC Public Health, 13, 17. 10.1186/1471-2458-13-17 23302300 PMC3546846

[bibr64-10436596261430091] Richardson A. Yarwood J. Richardson S. (2017). Expressions of cultural safety in public health nursing practice. Nursing Inquiry, 24(1), Article e12171. 10.1111/nin.12171 27905177

[bibr65-10436596261430091] Sandstrom H. Benatar S. Peters R. Genua D. Coffey A. Lou C. Adelstein S. Greenberg E. (2020). Home visiting career trajectories: Final report (OPRE report #2020-11). Office of Planning, Research, and Evaluation, Administration for Children and Families, U.S. Department of Health and Human Services. https://www.urban.org/sites/default/files/publication/101641/home_visiting_career_trajectories_0.pdf

[bibr66-10436596261430091] Schickedanz A. Escarce J. J. Halfon N. Sastry N. Chung P. J. (2021). Intergenerational associations between parents’ and children’s adverse childhood experience scores. Children (Basel, Switzerland), 8(9), 747. 10.3390/children8090747 34572179 PMC8466272

[bibr67-10436596261430091] Segal L. Sara Opie R. Dalziel K. (2012). Theory! The missing link in understanding the performance of neonate/infant home-visiting programs to prevent child maltreatment: A systematic review. The Milbank Quarterly, 90(1), 47–106. 10.1111/j.1468-0009.2011.00655.x 22428693 PMC3385020

[bibr68-10436596261430091] Selvaraj K. Korpics J. Osta A. D. Hirshfield L. E. Crowley-Matoka M. Bayldon B. W. (2022). Parent perspectives on adverse childhood experiences & unmet social needs screening in the medical home: A qualitative study. Academic Pediatrics, 22(8), 1309–1317. 10.1016/j.acap.2022.08.002 36007805

[bibr69-10436596261430091] Shaw D. S. Mendelsohn A. L. Morris P. A. (2021). Reducing poverty-related disparities in child development and school readiness: The Smart Beginnings tiered prevention strategy that combines pediatric primary care with home visiting. Clinical Child and Family Psychology Review, 24(4), 669–683. 10.1007/s10567-021-00366-0 34505232 PMC8428206

[bibr70-10436596261430091] Sukhera J. (2022). Narrative reviews: Flexible, rigorous, and practical. Journal of Graduate Medical Education, 14(4), 414–417. 10.4300/JGME-D-22-00480.1 35991099 PMC9380636

[bibr71-10436596261430091] Švorcová J. (2023). Transgenerational epigenetic inheritance of traumatic experience in mammals. Genes, 14(1), Article 120. 10.3390/genes14010120 PMC9859285 36672861

[bibr72-10436596261430091] Szyf M. (2015). Nongenetic inheritance and transgenerational epigenetics. Trends in Molecular Medicine, 21(2), 134–144. 10.1016/j.molmed.2014.12.004 25601643

[bibr73-10436596261430091] Taylor J. Novoa C. Hamm K. Phadke S. (2019, May). Eliminating racial disparities in maternal and infant mortality: A comprehensive policy blueprint. Center for American Progress. https://www.americanprogress.org/issues/women/reports/2019/05/02/469186/eliminating-racial-disparities-maternal-infant-mortality/

[bibr74-10436596261430091] von Bertalanffy L . (1969). General system theory: Foundations, development, applications. George Braziller.

[bibr75-10436596261430091] Walsh D. McCartney G. Smith M. Armour G. (2019). Relationship between childhood socioeconomic position and adverse childhood experiences (ACEs): A systematic review. Journal of Epidemiology and Community Health, 1979(12), 731087–731093. 10.1136/jech-2019-212738 PMC6872440 31563897

[bibr76-10436596261430091] Weiner B. (1985). An Attributional theory of achievement motivation and emotion. Psychological Review, 92(4), 548–573. 10.1037/0033-295X.92.4.548 3903815

[bibr77-10436596261430091] World Health Organization. (2025). Social determinants of health. https://www.who.int/health-topics/social-determinants-of-health#tab=tab_1

[bibr78-10436596261430091] Yehuda R. Lehrner A. (2018). Intergenerational transmission of trauma effects: Putative role of epigenetic mechanisms. World Psychiatry, 17(3), 243–257. 10.1002/wps.20568 30192087 PMC6127768

[bibr79-10436596261430091] Zhang L. Mersky J. P. Gruber A. M. H. Kim J.-Y. (2023). Intergenerational transmission of parental adverse childhood experiences and children’s outcomes: A scoping review. Trauma, Violence & Abuse, 24(5), 3251–3264. 10.1177/15248380221126186 36205317

[bibr80-10436596261430091] Zhang W. Rodziewicz G. Voss M. Lane S. (2022). Historical trauma and epigenetics. In Scrimshaw S. Lane S. Rubinstein R. Fisher J. (Eds.), The SAGE handbook of social studies in health and medicine (pp. 197–215). SAGE. https://www.worldcat.org/title/sage-handbook-of-social-studies-in-health-and-medicine/oclc/1286931854

